# Pleiotropic Effects of Tetracyclines in the Management of COVID-19: Emerging Perspectives

**DOI:** 10.3389/fphar.2021.642822

**Published:** 2021-04-23

**Authors:** Hayder M. Al-kuraishy, Ali I. Al-Gareeb, Mohammed Alqarni, Natália Cruz-Martins, Gaber El-Saber Batiha

**Affiliations:** ^1^ Department of Clinical Pharmacology and Medicine, College of Medicine, Al-Mustansiriya University, Baghdad Iraq; ^2^ Department of Pharmaceutical Chemistry, College of Pharmacy, Taif University, Taif, Saudi Arabia; ^3^ Faculty of Medicine, University of Porto, Porto, Portugal; ^4^ Institute for Research and Innovation in Health (i3S), University of Porto, Porto, Portugal; ^5^ Laboratory of Neuropsychophysiology, Faculty of Psychology and Education Sciences, University of Porto, Porto, Portugal; ^6^ Department of Pharmacology and Therapeutics, Faculty of Veterinary Medicine, Damanhour University, Damanhour, Egypt

**Keywords:** coronavirus disease 2019, acute respiratory distress syndrome, SARS-CoV-2, COVID-19, tetracyclines

## Abstract

Coronavirus disease 2019 (COVID-19) is a global infectious disease caused by severe acute respiratory syndrome coronavirus 2 (SARS-CoV-2). Approximately 15% of severe cases require an intensive care unit (ICU) admission and mechanical ventilation due to development of acute respiratory distress syndrome (ARDS). Tetracyclines (TCs) are a group of bacteriostatic antibiotics, like tetracycline, minocycline, and doxycycline, effective against aerobic and anaerobic bacteria as well as Gram-positive and Gram-negative bacteria. Based on available evidences, TCs may be effective against coronaviruses and thus useful to treat COVID-19. Thus, this review aims to provide a brief overview on the uses of TCs for COVID-19 management. SARS-CoV-2 and other coronaviruses depend mainly on the matrix metalloproteinases (MMPs) for their proliferation, cell adhesion, and infiltration. The anti-inflammatory mechanisms of TCs are linked to different pathways. Briefly, TCs inhibit mitochondrial cytochrome c and caspase pathway with improvement of lymphopenia in early COVID-19. Specifically, minocycline is effective in reducing COVID-19–related complications, through attenuation of cytokine storm as apparent by reduction of interleukin (IL)-6, IL-1, and tumor necrosis factor (TNF)-α. Different clinical trials recommend the replacement of azithromycin by minocycline in the management of COVID-19 patients at high risk due to two main reasons: 1) minocycline does not prolong the QT interval and even inhibits ischemia-induced arrhythmia; 2) minocycline displays synergistic effect with chloroquine against SARS-CoV-2. Taken together, the data presented here show that TCs, mainly doxycycline or minocycline, may be potential partners in COVID-19 management, derived pneumonia, and related complications, such as acute lung injury (ALI) and ARDS.

## Introduction

Coronavirus disease 2019 (COVID-19) is a global infectious disease, actually considered a pandemic by the WHO, caused by severe acute respiratory syndrome coronavirus 2 (SARS-CoV-2). SARS-CoV-2 is a positive-sense, single-strand RNA virus sharing a genetic similarity with other *Betacoronaviruses*, such as Middle East respiratory syndrome coronavirus 1 (MERS-CoV-1) and severe acute respiratory syndrome coronavirus 1 (SARS-CoV-1) (Al-Kuraishy et al., 2020a). Specifically, SARS-CoV-2 binds to a specific receptor, called angiotensin converting enzyme 2 (ACE2) receptors, which are highly expressed in lung epithelial cells, proximal renal tubules, the heart, and even the brain. ACE2 is considered as the main target of the virus. SARS-CoV-2 infection triggers an acute host immune response, inflammatory reaction, and cytokine storm, leading to acute lung injury (ALI) and acute respiratory distress syndrome (ARDS) (Al-kuraishy et al., 2020b).

The genomic analysis of SARS-CoV-2 shows four structural proteins, namely, nucleocapsid protein (NP), membrane protein (MP), envelop protein (EP), and spike protein (SP), and four nonstructural proteins, namely, 3-chymotrypsin-like proteins (3CLpro), papain-like protease (PLpro), helicase, and RNA polymerase (Ser et al., 2020). Both PLpro and 3CLpro are involved in SARS-CoV-2 replication and transcription, with these proteins’ inhibition leading to a significant suppression of viral replications. However, 3CLpro is regarded as the main protease (Mpro), which concerns to all steps of SARS-CoV-2 life cycle. Thus, Mpro inhibitors may be effective against COVID-19 through inhibition of SARS-CoV-2 replication (Forster et al., 2020).

The clinical presentation and spectrum of COVID-19 varies from asymptomatic to mild–moderate clinical forms, with severe cases often needing hospitalization. Approximately 15% of severe cases require intensive care unit (ICU) admission and mechanical ventilation due to development of ARDS (Al-Kuraishy et al., 2020a). At present, different treatment modalities and approaches have been proposed to treat COVID-19, but there is no strong clinical evidence of their efficacy and safety, as they derive from animal and *in vitro* studies or from previous experiences during MERS-CoV and SARS-CoV pandemics. More recently, different randomized and nonrandomized clinical trials have been adopted; besides, prospective studies are underway (Trivedi et al., 2020).

Tetracyclines (TCs) are a group of bacteriostatic antibiotics, including tetracycline, minocycline, and doxycycline, which have shown to be effective against aerobic and anaerobic bacteria as well as Gram-positive and Gram-negative bacteria, with exceptions of *Proteus* species and *Pseudomonas aeruginosa* strains, which have intrinsic resistance. TCs act through inhibition of charged aminoacyl-tRNA attachment on the 30 subunits of microbial ribosomes (Peiris et al., 2017). Depending on present and previous evidences, TCs may be effective against coronaviruses, including SARS-CoV-2. Indeed, SARS-CoV-2 and other coronaviruses depend mainly on matrix metalloproteinases (MMPs) for their proliferation, cell adhesion, and infiltration. Zinc is a corner stone of MMPs; therefore, zinc chelation by TCs may limit SARS-CoV-2 infection and COVID-19 development (Ohe et al., 2020). Recent data have reported that TCs have noteworthy antiviral effects against single-strand, positive-sense RNA viruses, like dengue virus and SARS-CoV-2, through inhibition of RNA polymerase and serine protease (Li et al., 2017). In addition, doxycycline reduces viral replication, inhibiting the viral entry into cultured cells, with a consequent reduction of viral load (Mosquera-Sulbaran and Hernández-Fonseca, 2021). Indeed, a previous study revealed that the combination of doxycycline and ribavirin is more effective against chikungunya virus (Ferreira et al., 2019). In this sense, this review aims to provide a brief overview on the potential role of repurposing TCs in the management of COVID-19 that requires comprehensive evidences before their use in clinical practice.

## Tetracyclines and COVID-19

TCs have shown great potential for the management of COVID-19 through inhibition of SARS-CoV-2–induced hyperinflammation and cytokine storm, since TCs downregulate the production of inflammatory cytokines, including interleukins (IL-6, IL-33, and IL-1β) and tumor necrosis factor (TNF)-α. Also, doxycycline inhibits the expression of CD26 and CD147, which are important entry points for SARS-CoV-2 (Ohe et al., 2020). A study conducted by Yates et al. (2020) involving four case series of high-risk COVID-19 patients who were placed on doxycycline therapy for 5–14 days illustrated clinical and radiological improvements following 14 days of doxycycline therapy. Despite the safety profile of doxycycline, the authors do not recommend the general use of this drug for treating COVID-19 patients, except if under direct supervision and monitoring by physician.

Bonzano et al. (2020) confirmed that administration of doxycycline 200 mg/day in six COVID-19 patients with anosmia and other respiratory symptoms for 8 days led to rapid recovery of smell within 2 days, with the average time of 2.5 ± 0.5 days. This improvement was related to the modulation of ACE2 and CD147 expressions at the olfactory epithelium.

It has been shown that SARS-CoV-2–induced hyperinflammation is linked to mast cell proliferation and stimulation at the respiratory submucosa with subsequent release of histamine, IL-1, and IL-33. Also, TCs and their derivative inhibit proliferation with induction of mast cell apoptosis and activation of protein kinase C, ultimately inhibiting respiratory inflammation and cytokine storm (Alam et al., 2020). Moreover, both minocycline and doxycycline are effective in attenuating COVID-19–induced ARDS and cytokine storm (Mostafa, 2020). Similarly, by virtue of their lipophilic properties, TCs have a higher penetration to the basement membrane of alveolar cells and may also efficiently cross the SARS-CoV-2 envelope (Mostafa, 2020). Therefore, the use of TCs seems promising in the management of COVID-19–derived pneumonia due to their ability to inhibit SARS-CoV-2 replication and associated inflammatory reactions. Furthermore, TCs are safer than chloroquine and antiretroviral drugs that are commonly and initially used in the management of COVID-19 pneumonia (Poinas et al., 2020).

Zhao and Patankar (2020) also found that TCs and doxycycline are more effective than either chloroquine or doxycycline in inhibiting SARS-CoV-2 binding to the ACE2, as they are able to inactivate the viral receptor-binding domain. In the same way, Bharadwaj et al. (2020), in a molecular docking computational study, found that TCs are effective against SARS-CoV-2 through inhibition of membrane protein (Mpro). Based on combinatorial molecular simulation analysis, doxycycline and minocycline revealed to be potent inhibitors of SARS-CoV-2 Mpro, and therefore can be used in combinational therapy against SARS-CoV-2 infection. Moreover, different *in vitro* studies illustrated that doxycycline has anti-SARS-CoV-2 activity with suppression of bacterial coinfections and associated inflammatory changes (Gendrot et al., 2020). Previous experimental studies showed that minocycline was effective against Japanese encephalitis virus through modulation of microglial activation, neural apoptosis, and viral replication (Mishra and Basu, 2008).

In addition, a recent retrospective multi-institutional cohort study illustrated that within a year, TC users have low risk of ARDS, shorter stay length in the ICU, and lower need for mechanical ventilation. Both doxycycline and minocycline and other TCs exert potent anti-inflammatory effects as they inhibit the proliferation of T-cells and inflammatory cytokines with subsequent suppression of the development of ARDS. This finding indicates the prophylactic role of TCs in the prevention of COVID-19–induced ARDS. Also, TCs downregulate the CD40 ligand on T-cells and prevent lung inflammation progression during COVID-19 pneumonia (Byrne et al., 2020). Alam et al. (2020), in an observational study, examined 89 patients with COVID-19 from March to May 2020 and showed that early treatment with doxycycline (10 mg/day) for 7 days in high-risk nonhospitalized COVID-19 patients was linked to an early clinical recovery and decreased hospitalization and mortality.

## Anti-Inflammatory Effects of Tetracyclines in COVID-19

As stated above, the anti-inflammatory mechanisms of TCs are linked to different pathways at both cellular and molecular levels. Briefly, TCs inhibit mitochondrial cytochrome c and caspase-1 pathway with improvement of lymphopenia in early COVID-19 cases (Moullan et al., 2015). In COVID-19, invasion of alveolar epithelial type II (AEC-II) cells by SARS-CoV-2 causes inflammatory reactions through activation of nuclear factor kappa-light-chain enhancer of the activated B-cell pathway (NFκB). The activated NFκB pathway during COVID-19 leads to AEC-II cell apoptosis with reduction of alveolar surfactant and downregulation of ACE2, while it prolongs neutrophil survival and accumulation (Hariharan et al., 2020). Also, the activated NFκB pathway increases the differentiation and response of the pro-inflammatory macrophage phenotype, which per se causes cytokine storm and further NFκB activation in a vicious cycle (Carcaterra and Caruso, 2021). Kircheis et al. (2020) revealed that inhibition of the NFκB pathway during SARS-CoV-2 infection might be a potential target in attenuating the cytokine storm progression through inhibition of pro-inflammatory cytokines, such as IL-6, IL-1, TNF-α, chemokines, and adhesion molecules. Different inhibitors of the NFκB pathway, such as dexamethasone and acetylsalicylic acid, are also able to reduce the cytokine storm in severe COVID-19 and other viral infections (Neufeldt et al., 2020). Also, it has been reported that the anti-inflammatory effect of TCs is related to their ability to suppress NFκB transcription factor, through inhibition of IκBα (IKK) activation and nuclear translocation of NFκB (Sun et al., 2015). In COVID-19, TCs downregulate the NFκB pathway and other inflammatory signaling pathways, such as p38, ERK1, and MAPK pathways, with significant inhibition of SARS-CoV-2 main protease (Mosquera-Sulbaran and Hernández-Fonseca, 2021).

These changes prevent T-cell suppressor apoptosis, which ultimately averts the exacerbation of immune activations in late COVID-19 pneumonia. Moreover, the apoptosis inhibition may stop endothelial dysfunction–induced microthrombosis (Ferreira et al., 2020). Also, TCs preclude neutrophil migration, chemotaxis, release of pro-inflammatory cytokines, local oxidative stress, and vascular leakage. Thus, TCs may prevent COVID-19–induced ARDS (Byrne et al., 2020). Specifically, doxycycline inhibits inflammation-induced lymphangiogenesis through attenuation of vascular endothelial growth factor signaling, thus attenuating neovascularization-induced alveolar hemorrhage, alveolar collapse, and refractory hypoxemia in COVID-19 pneumonia (Connors and Levy, 2020).

On the other hand, Portnoy et al. (2020) found that activation of MMPs, mainly MMP-2 and MMP-9, by SARS-CoV-2 leads to the degradation of alveolar basement membrane. Briefly, MMPs are synthesized and released from macrophages and neutrophils involved in ALI (Ueland et al., 2020). In addition, it has been shown that macrophage activation and infiltration is associated with SARS-CoV–induced ALI. Also, COVID-19 pneumonia is associated with macrophage activation syndrome with elevation of IL-6 levels. Therefore, MMP inhibition by TCs might explain the beneficial role of such agents in controlling COVID-19–induced ARDS as evident by the reduction of IL-6 serum levels (McGonagle et al., 2020). Recent data have shown that minocycline is an effective drug, being able to reduce COVID-19–related complications through attenuation of cytokine storm as apparent by the reduction of IL-6, IL-1, and TNF-α (Singh et al., 2020). Previously, Di Caprio et al. (2015) reported that a low dose but not large-dose of doxycycline is effective in the inhibition of inflammatory reactions in chronic inflammatory disorders. Thus, based on this evidence, minocycline and doxycycline seem to be promising drugs for COVID-19 therapy; however, different preclinical and clinical studies are warranted before the final recommendation of minocycline for COVID-19 treatment, due to reduction of all steps of inflammation (Carcaterra and Caruso, 2021).

On the other hand, but also worthy of note is that as chloroquine is extensively used alone or in combination with azithromycin in the management of COVID-19–derived pneumonia, the risk of QT prolongation and arrhythmias is increased, mainly in males and elderly patients, which represent the main proportion of COVID-19 population (Diana et al., 2020). Moreover, Wang et al. (2020) disclosed that 44.4% of hospitalized COVID-19 patients and 16.7% of those in ICU have arrhythmias due to electrolyte disturbances, hypoxia, and use of chloroquine alone or in combination with azithromycin. Therefore, various clinical studies have recommended the replacement of azithromycin by minocycline in the management of high-risk COVID-19 patients for two main reasons: 1) Minocycline does not prolong the QT interval and even inhibits ischemia-induced arrhythmia; 2) minocycline has a synergistic effect with chloroquine against SARS-CoV-2 (Diana et al., 2020). Therefore, early treatment with TCs, mainly doxycycline or minocycline, for high-risk COVID-19 patients seems linked to an early recovery, reduced hospitalization, and decreased mortality rate. Oliveira et al. (2020), in a clinical trial study, demonstrated that lopinavir plus doxycycline is more effective in the management of COVID-19 patients due to a synergistic inhibition of SARS-CoV-2 protease. Nevertheless, Cag et al. (2020) showed that combination of minocycline and chloroquine offers many benefits in the management of moderate-to-severe COVID-19 patients, since both drugs are widely available and cheap, and their contraindications are well-known and recognized. As well, the anticytokine and antimicrobial effects of this combination may mitigate both morbidity and mortality rates among COVID-19 patients alternative to the costlier drugs.

Regarding other modalities in the management of severe COVID-19 patients, tocilizumab (IL-6 antagonist) has been used to overcome the cytokine storm and development of ALI and ARDS. However, there are limited real-life data regarding the effect of tocilizumab in COVID-19 management (Gautam et al., 2020). A retrospective study showed that tocilizumab therapy may reduce the need for mechanical ventilation and death in patients with severe COVID-19 (Luo et al., 2020). However, Guaraldi et al. (2020) confirmed that tocilizumab was not effective in preventing intubation or death in hospitalized COVID-19 patients. Besides, unlike TCs, tocilizumab is an expensive drug that also needs close monitoring during its parenteral administration since it may cause serious anaphylactic reactions, liver injury, and secondary bacterial infections (Stone et al., 2020). On the other hand, TCs are cheap, safe, and effective orally, not needing close monitoring and neither increasing the risk of bacterial coinfection; therefore, TCs are more effective than tocilizumab in the management of COVID-19 (Gatti et al., 2020).

Moreover, TCs could be helpful in another therapeutic situation rarely occurring in COVID-19 patients, that is, the treatment of bacterial superinfections. A recent report showed that superinfections sustained by atypical bacteria, such as *Mycoplasma* intracellular pathogens, seem to be the most frequent ones (Huang et al., 2020). Incidentally, TCs similar to azithromycin but with no cardiological issues are highly active against these bacteria which, on the contrary, are naturally insensitive to beta-lactams (Sodhi and Etminan, 2020; McKenna, 2020). This, along with their broad spectrum, could suggest using TCs as a treatment eventually in association with beta-lactams or other antibiotics in the empirical management of superinfections (Conforti et al., 2020). Also, an indiscriminate use of TCs in the absence of bacterial superinfections is in conflict with the recent antimicrobial stewardships in such a way that its use, although strictly related to COVID-19 patients, should be carefully evaluated under a risk/benefit light (Chakraborti et al., 2020).

Likewise, secondary bacterial infections in hospitalized COVID-19 patients with pneumonia are infrequent due to frequent empirical antibiotic treatment (Bhowmik et al., 2020). Langford et al. (2020) found that SARS-CoV-2 is often linked to Gram-positive and Gram-negative bacterial infections, similar to atypical bacteria that can cause bacterial pneumonia, ultimately increasing the risk of mortality and complications. Thus, TCs might be effective in the management of COVID-19 pneumonia due to their experimental anti-SARS-CoV-2 effects and broad-spectrum antibacterial activity (Mirzaei et al., 2020).

## Tetracyclines and COVID-19 Complications

Most SARS-CoV-2 infections are mild; however, in severe cases, they are associated with systemic complications due to propagation of cytokine storm, dysregulation of the renin–angiotensin NLRP3 system (RAS), and activation of Nod-like receptor pyrin 3 (NLRP3) inflammasomes (Manna et al., 2020). The activation of NLRP3 inflammasomes and toll-like receptor 4 (TLR4) by SARS-CoV-2 or direct SARS-CoV-2 invasion through ACE2 receptors, which are expressed in different tissues, may lead to various organ injuries (Polidoro et al., 2020). Among such SARS-CoV-2 infection-derived complications, acute cardiac injury, and neurological, hematological, endocrinological, and metabolic complications appear to be the most frequently observed and are collectively called as extrapulmonary manifestations of COVID-19 (Van den Berg and Te Velde, 2020).

**FIGURE 1:**
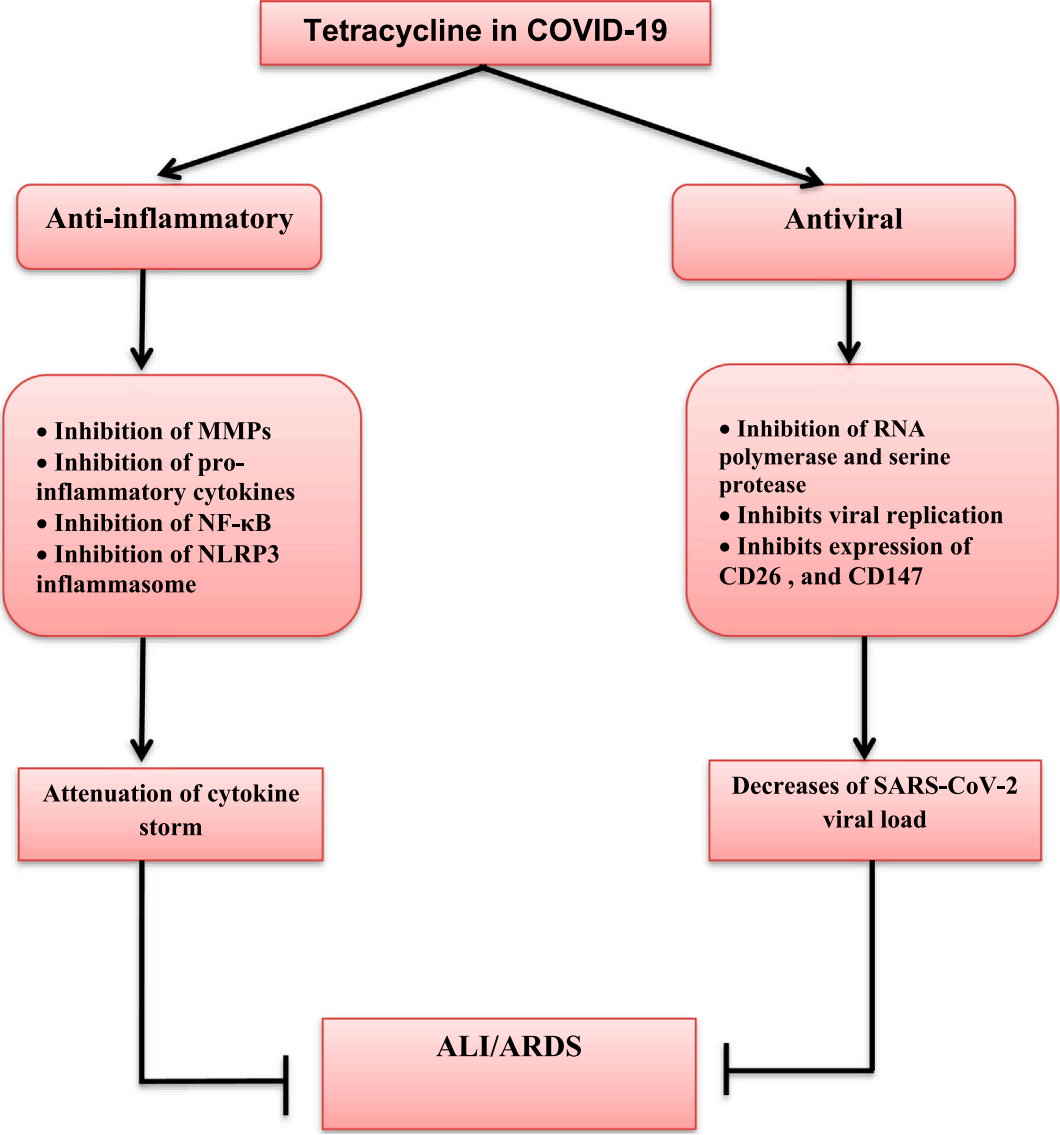
Pleiotropic effects of tetracycline in SARS-CoV-2 infection. Tetracycline has antiviral and anti-inflammatory effects. Antiviral effects of tetracycline are through inhibition of RNA-polymerase and serine protease dependent viral replications, and inhibition of the expression of CD26 and CD147, which are regarded as entry-point for SARS-CoV-2 with subsequent reduction in viral load. Anti-inflammatory effects of tetracycline are through inhibition of matrix metalloproteinases (MMPs), nuclear factor kappa B (NF-κB), Nod-like receptor pyrin 3 (NLRP3) inflammasome, and release of pro-inflammatory cytokines with subsequent attenuation of cytokine storm development. Taken together, both anti-inflammatory and antiviral effects of tetracycline inhibit development of acute lung injury (ALI) and acute respiratory distress syndrome (ARDS) in COVID-19.

The most common COVID-19 complications are ARDS and acute cardiac injury, although they can be mitigated by TC therapy. Gupta et al. (2020), in an experimental mice model, showed that tetracycline reduces the risk of ALI and ARDS by inhibition of NLRP3 inflammasomes. Moreover, doxycycline inhibits MMP2-mediated degradation of myosin light chain 1 and troponin during experimental myocardial ischemic-reperfusion injury (Bode et al., 2019). Besides, minocycline has a cardioprotective effect during myocardial ischemic-reperfusion injury through the inhibition of NFκB activation and pro-inflammatory cytokine release (Bil-Lula et al., 2018). Hence, through their anti-inflammatory and immunomodulatory effects, TCs lead to a reduction of both systemic and life-threatening complications of COVID-19 (Yi et al., 2019). Also, doxycycline and other type of TCs may reduce sepsis-induced cytokine storm and associated systemic organ damage during severe secondary bacterial infection in COVID-19 patients (Sargiacomo et al., 2020). Therefore, the net pleiotropic effect of tetracycline is summarized in Figure 1.

Nonetheless, also worthy of note is that the primary concern while using TCs in COVID-19 management is related to their contraindications in children, sulfa drug allergy, pregnancy, and lactation. Indeed, serious side effects, such as photosensitivity, drug-induced hepatitis, and erythema multiforme, may occur and thus should be evaluated prior to the use of TCs (Patel et al., 2020). In addition, COVID-19 may be linked to skin manifestations such as red itchy patches and itchy blisters that result from direct SARS-CoV-2 invasion, inflammatory reactions, and allergic reactions to repurposing drugs, such as TCs (Hamblin and Abrahamse, 2019).

## Conclusion

Taken together, data presented here highlight that the antiviral, anti-inflammatory, immune-modulatory, and cardioprotective abilities of TCs, mainly doxycycline or minocycline, make them potential partners in the management of COVID-19–derived pneumonia and related complications, such as ALI and ARDS. In addition, both doxycycline and minocycline have noteworthy therapeutic and prophylactic effects against SARS-CoV-2 infection.
